# The psycho-social impact of obstetric fistula and available support for women residing in Nigeria: a systematic review

**DOI:** 10.1186/s12905-023-02220-7

**Published:** 2023-02-25

**Authors:** Ifunanya Roseline Nduka, Nasreen Ali, Isabella Kabasinguzi, David Abdy

**Affiliations:** Institute of Health Research, University of Bedfordshire, Luton, LU1 3JU UK

**Keywords:** Obstetric fistula, Psycho-social impact, Support, Rehabilitation, Reintegration, Women, Nigeria

## Abstract

**Background:**

Obstetric fistula continues to affect the social and mental wellbeing of women living in Nigeria giving rise to poor maternal health outcome. While the World Health Organisation (WHO) has recommended the introduction of psycho-social interventions in the management of obstetric fistula women, psycho-social support for women living with obstetric fistula in Nigeria, are limited. This systematic review aimed to synthesise the psycho-social impact of obstetric fistula on women living in Nigeria as well as the available psycho-social support for these affected women.

**Methods:**

Following a keyword strategy, Medline, CINAHL, Google scholar, ScienceDirect, Cochrane library, PsychINFO, AMED, British Nursing database, Pubmed central, TRIP database, UK Pubmed central, socINDEX, Annual reviews, ISI Web of Science, Academic search complete, Credo reference, Sage premier and Scopus databases were searched alongside hand searching of articles. The inclusion criteria were set as articles published between 2000 and 2020, on the psychosocial consequences of obstetric fistula in Nigeria. The Critical Appraisal Skills Program (CASP) tool was used to appraise the quality of the included studies. The data was extracted and then analysed using narrative synthesis.

**Results:**

620 relevant citations were identified, and 8 studies were included. Women with obstetric fistula, living in Nigeria were found to be ostracised, abandoned by families and friends, stigmatised and discriminated against, which led to depression, loneliness, loss of self-esteem, self-worth and identity. Psycho-social interventions for women who experienced obstetric fistula are not widely available.

**Conclusion:**

There is a need for the introduction of more rehabilitation and reintegration programs across the country. The psychosocial effect of obstetric fistula is significant and should be considered when developing interventions. Further, more research is needed to evaluate the sustainability of psychosocial interventions in Nigeria.

## Background

Obstetric fistula is the constant leakage of urine, faeces or both due to injury caused during childbirth [1–3]. Obstetric fistula occurs when a woman’s pelvis is too narrow for the foetus to pass through, and the labour prolongs due to lack of access to emergency obstetric care to discharge the obstruction [4]. During prolonged labour, the foetal head exerts a long consistent pressure on the pelvis which disturbs the circulation of blood in the soft tissues surrounding the woman’s vagina, rectum and bladder [4, 5]. The United Nations Population Fund (UNFPA) estimated that Nigeria accounts for 12,000 new cases annually with a current estimated total of 150,000 cases [6]. The actual prevalence of obstetric fistula may be higher due to the under-reporting of cases in Sub-Saharan Africa [7].

Obstetric fistula affects a woman’s physical, economic, psychological and social life [1, 8]. Women living with obstetric fistula suffer urogenital and kidney infection, severe skin irritation and genital lacerations due to incontinence [7, 9]. Aside these, their quality of life is also deeply affected as they face discrimination and stigma from family, friends and the community at large [7, 9]. As a result, they become isolated and are unable to be involved in family, religious and social gatherings [3, 7]. Separation and divorce are also common among women living with obstetric fistula, leaving them lonely [3, 4, 7].

Most women with obstetric fistula lose their source of income due to their incontinence and become an economic burden to their families [1, 10]. This pushes them to poverty, leaving them in bitterness, trauma, depression and suffering disappointment [9]. The lack of support and care from families and the community, the physical and economic incapacity alongside social stigma accompanied with obstetric fistula has affected the quality of life of the affected women and led to multiple suicide attempts [1, 11].

Nonetheless, with the use of adequate and standard resources and effective surgical practice, obstetric fistula can be treated with a closure rate of 80–90% [12]. Sadly, due to lack of resources and skilled surgeons in Nigeria, most affected women are bound to live with the condition for a long time or throughout the course of their lives [2, 12, 13]. Also, other factors such as poor transport system, poor access to perinatal care which are factors that arise where these women live, could prevent them from seeking surgery [14]. Furthermore, given the severe psycho-social consequences of obstetric fistula, studies have shown that surgical repair is not enough in the care of obstetric fistula [11, 15]. Psycho-social support for these women is also needed to reintegrate them into the community.

The mental health impact of living with obstetric fistula is now well recognised and reported [8, 16, 17]. The WHO has recommended the inclusion of psychosocial support in the care of obstetric fistula [18]. However, there is dearth of evidence reporting the psycho-social interventions addressing the impact of obstetric fistula on women living in Nigeria [6, 8]. Also, much attention has not been directed towards the psycho-social outcomes of obstetric fistula even when the evidence base suggests severe psychological and social outcomes for women with obstetric fistula [1, 8, 12, 19, 20]. Some studies have reviewed the general consequences of obstetric fistula [20, 21] but this review shed more light on the psychosocial impacts. This study aimed to review the existing literature on the psycho-social impact of obstetric fistula and the interventions, appraise the available evidence related to psycho-social support for obstetric fistula women living in Nigeria. Synthesising the existing evidence can potentially identify the gaps in the available literature and identify areas for further consideration.

## Methods

### Inclusion and exclusion criteria

Book chapters, case reports, grey literature and peer-reviewed journal articles with primary data from Nigeria, published in English language, that studied women residing in Nigeria**,** reported the psycho-social impact of obstetric fistula, psycho-social support for obstetric fistula women and was conducted after the year 2000, were eligible for selection. Studies not published in English language and peer-reviewed journals, including secondary data, not reporting the psycho-social impact of obstetric fistula on women residing in Nigeria, published before the year 2000 were excluded.

### Search strategy

Boolean operators such as “AND” and “OR” were used alongside the keywords to search for studies in the following electronic databases: Medline, CINAHL, Google scholar, ScienceDirect, Cochrane library, PsychINFO, AMED, British Nursing database, Pubmed central, TRIP database, UK Pubmed central, socINDEX, Annual reviews, ISI Web of Science, Academic search complete, Credo reference, Sage premier and Scopus, in June 2020. The keywords used include: (psycholog* OR social OR mental OR depress* OR psycho-social OR “lived experience”) AND (support OR Intervention OR rehabilitation OR reintegration) AND (consequence OR impact OR effect) AND (obstetric fistula OR fistula OR Vesicovaginal) AND (maternal OR women) AND (resident OR living) AND (Nigeria OR Africa OR developing world). The titles and abstracts of the studies selected from the search were screened against the set inclusion and exclusion criteria. Additional studies were found from hand searching and screening the reference lists of already identified studies.

### Data extraction

Data extraction was performed by one author. Once the selected studies were obtained, data were extracted independently into a Microsoft Excel spread sheet. The headings of the data extraction spreadsheet included the author, date of publication, aim of study, study setting, study design, participant characteristics, sampling method, study size, data collection and main findings. Other findings that did not fit into the pre-defined headings were collated in a different column and analysed separately.

### Quality appraisal

The risk of bias and rigor of the selected studies were assessed using the Critical Appraisal Skills Programme (CASP).. The CASP tool is used to appraise quantitative, qualitative and mixed methods research. It is made up of checklists on appraising each research method and each checklist contains 10 questions [22]. Out of the 10 questions; 9 questions addressed quality while 1 question addressed value. The quality appraisal of the studies followed a scoring system which was graded as either low (1–3 scores), moderate (4–6 scores) or high (7–10 scores) quality. Nonetheless, studies were not excluded by level of quality, in accordance with other qualitative reviews [23, 24].

### Analysis

The results were analysed using narrative synthesis. This approach was used due to the heterogeneity of the selected studies in this review. Evidence has reported the usefulness of narrative analysis in discovering the core ideologies rooted in stories and cultural values [25]. Thus, narrative synthesis was used to effectively interpret the data.

## Results

**Fig. 1:**
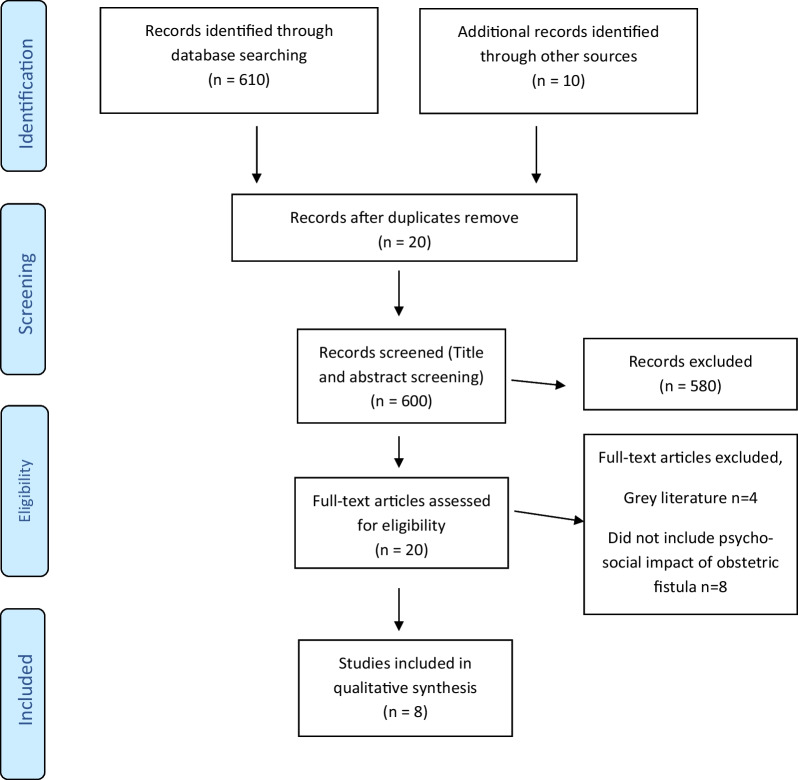
PRISMA flow diagram of the study selection process

A total of 620 studies were identified from the electronic searches (n = 610) and hand searches (n = 10). Twenty articles passed the title and abstract screening. However, only 8 articles met the inclusion and exclusion criteria after a thorough full-text screening and was included in the review. The study selection process is shown in the PRISMA flow diagram in Fig. 1.

### Quality appraisal

The CASP checklist identified only one study [3], to be of high-quality (9 scores), three studies [9, 26, 27] to be of moderate quality (4–6 scores) while 4 studies [7, 28–30] were of low quality (2–3 scores) due to shortcomings in their methodologies and data analysis. Although all studies obtained ethical approval from the ethics committee, only two studies [3, 27] provided sufficient details of how the research was explained to the participants to assess if ethical standards were maintained. Further, none of the studies explained if the relationship between the researcher and the participants were sufficiently considered [31] which compromised the quality of the studies.

### Demographic features of participants

All participants of the selected studies were women with obstetric fistula who were receiving treatment from healthcare centres except one [3] whose participants were women who were previously living with obstetric fistula, had undergone obstetric fistula repair, rehabilitated, and returned to their communities. The ages of the women were recorded by all selected studies which was an average of 37 years. All studies reported the educational qualifications of the women with majority having no formal education. In addition, all studies recorded the employment status of the women except for two studies [28, 29]. While some of the women were unemployed [3, 9, 26, 30], some women were farmers and artisans [3, 7, 26, 27], some were traders [30] while some were students [3, 26]. The background, methodological details and key findings of the included studies are shown in Table 1.

**Table 1:** Background, methodological details and key findings of the included studies

S. No	Author (s) and year of publication	Location of study	Research objectives	Study design and time	Sample method of recruitment and participants’ characteristics	Main findings	Quality rank
1	Kabir et al*.* (2003)	Muritala Muhammed Specialist Hospital, Kano	To assess the consequences of vesico-vaginal fistula among women presented with vesico-vaginal fistula	Cross-sectional study, use of structured questionnaire; November to December 2001	120 women with vesico-vaginal fistula were sampled but the sampling technique was not specified. The age distribution of the women includes 10–15 (n = 98), 16–20 (n = 18), 20–25 (n = 4)	More than half of the women (n = 61) were bitter about their condition, almost half of the women (n = 40) were depressed while a minority of the women (n = 9) were indifferent No psycho-social support was reported	Low
2	Hassan and Nasir (2019)	Maryam Abacha Fistula Hospital, Sokoto	To determine the frequencies of the common comorbidities in patients managed for obstetric fistula	Prospective cross-sectional study; use of Beck’s inventory	A total of 179 patients including married women (n = 155), widows (n = 4) and divorcees (n = 20) were sampled. Their age distribution comprised of 14–19 (n = 50), 20–24 (n = 43), 25–29 (n = 27) and more than 30 years (n = 59). A total of 92.7% (n = 166) had no formal education, 4.5% (n = 8) had just primary education while 2.8% (n = 5) had secondary education. However, the method of recruiting the participants was not stated	A total of 106 (59.2%) had foot drop and approximately 58.1% of the participants (n = 104) were reported to be depressed. No psycho-social support was recorded	Low
3	Nsemo (2014)	Family Life Centre and Hospital, Mbribit Itam, Akwa Ibom state	To assess the extent to which abandonment, social isolation and stigmatisation significantly influence the coping strategies of women affected by vesicovaginal fistula	Cross-sectional study with the use of structured and unstructured interview. Time of the research was not recorded	A total of 120 women seeking treatment from the obstetric fistula centre were sampled through purposive sampling. Majority of the women (n = 80) were divorced while 27.50% (n = 33) were still married. Most of the women (n = 40) were primary school leavers while about 31.79% (n = 38) stopped at secondary education	While a minority of the women received support from their families and spouse, most of the women were abandoned and rejected by their spouses. While some of the women were stigmatised, some were filled with fear. Some of the women were isolated from their co Also, the abandonment and stigmatisation they experienced negatively affected their coping abilities	Moderate
4	Nweke and Igwe (2017)	National Obstetric Fistula Centre, Abakaliki, Ebonyi state	To explore the psycho-social experiences of patients with vesicovaginal fistula	Cross-sectional study; focus group discussions. No time frame was recorded	100 women were sampled. Majority of the women (n = 95) were rural dwellers while more than half of the women (n = 54) were without a formal education. The age distribution included 16–25 (n = 14), 26–35 (n = 40), 36–45 (n = 27), 45 years and more (n = 19). The sampling technique used was not recorded	Psycho-social impact on the participants included helplessness, sadness, suicidal thoughts, stigma and blame, feeling of worthlessness, fear of divorce, shame and social withdrawal and perinatal loss. Social support received by a minority of the women (n = 33) was recorded	Low
5	Fehintola et al*.* (2019)	Obstetric fistula unit of Obstetrics and Gynaecology department of Obafemi Awolowo University Teaching Hospital Complex, Ilesha, Osun state	To examine the medical and psycho-social consequences of obstetric fistula on the patients	Cross-sectional study with structured interviews and focus group discussions between July 2017 to December 2018	A total of 86 patients with ages including 15–24 (n = 15), 25–34 (n = 40), 35–44 (n = 18), 45–54 (n = 10), more than 55 (n = 3) were sampled through purposive sampling	A divorce rate of 40% was reported among the patients. About 38.4% (n = 33) of the patients were depressed, 19.8% (n = 17) had lost their self-esteem, 75.6% (n = 65) felt ostracised, 52.3% (n = 45) were bitter and 37.2% (n = 32) were isolated due to shame. Infertility was recorded in 19.8% (n = 17) while perinatal loss was experienced in 85% of the respondents. Social support which included consolation and encouragement were reported in a few patients	Moderate
6	Degge et al*.* (2019)	Evangel Vesico-Vaginal Fistula (EVVF) centre, Bingham University Teaching Hospital, Jos	To understand the identities fistula survivors, ascribe to themselves and explore the impact of these identities through the process of their illness experiences	Narrative inquiry with an episodic narrative interview. No time frame recorded	15 women who were previously with obstetric fistula, undergone repair, rehabilitated and were re-integrating into their communities were recruited using purposive sampling	The women experienced shame, psychological trauma, loss of self-worth, despair, identity change, stigma, divorce, sense of hopelessness, suicidal thoughts. Rehabilitation program was reportedly available for women with obstetric fistula	High
7	Okoye, Emma-Echiegu and Tanyi (2014)	National Fistula Centre, Abakaliki, Ebonyi state	To explore the lived experiences of vesicovaginal fistula victims and their coping strategies	Cross-sectional study with taped interview in October 2010	10 women awaiting obstetric fistula repair were sampled through purposive sampling. The mean age of these women was 35 years. Only 3 of the women were still married while only 1 lacked formal education	The studied women were ostracised by their families, spouse and communities, shamed and depressed. Loss of self-esteem, perinatal loss, stress and anxiety were also reported among the respondents. Psycho-social support from families was reported	Low
8	Emma-Echiegu, Okoye and Odey (2014)	National Fistula Centre, Abakaliki, Ebonyi state	To examine the knowledge of causes of vesicovaginal fistula on vesicovaginal fistula patients and explore experiences of stigma and discrimination of vesicovaginal fistula	Cross-sectional study with focus group discussions between August and December 2011	30 women between the ages of 15 to 65 were sampled through purposive sampling. A total 33.3% (n = 10) had no formal education while only 20% (n = 6) attained secondary education. Most of the women (n = 14) were divorced while 13.3% (n = 4) were never married	Divorce, loss of child, depression, stigma, discrimination and social isolation were reported among the studied women. No psycho-social support was reported	Low

### The psycho-social impact of obstetric fistula on women living in Nigeria

The psycho-social impact of obstetric fistula was identified in all selected studies. Reported impacts were loss of marriage (divorce), depression, stigma, low self-esteem, discrimination andabandonment.In addition, social isolation, ostracization, feelings of worthlessness, suicidal thoughts, loss of identity, helplessness, sadness and loneliness were reported..

Five studies reported loss of marriage due to obstetric fistula among women [3, 7, 9, 26, 27]. According to three studies [3, 26, 27], women were separated or divorced from their husbands. Two studies attributed the loss of marriage to the feeling of shame on the part of the husband [7, 9]. Conversely, one study reported that the studied women with obstetric fistula were still with their husbands, although were in fear of losing their spouse [29]. Nonetheless, this study utilised a small sample size which may be a limitation.

Four studies identified abandonment and social isolation as consequences of obstetric fistula [9, 26, 27, 29]. While one study reported that the studied women with obstetric fistula were abandoned by just their spouse but supported by their natal families [27], two studies reported that these women were abandoned by their spouse and family [7, 26]. Respondents in Nsemo [26] were reported to be lonely. However, there were a few exceptions noted by Okoye, Emma-Echiegu and Tanyi [7] who were not abandoned by their families. Also, while some respondents in Nsemo [26] reported being abandoned, some respondents confessed to being supported by their husband and family. All four studies attributed the cause of social isolation to stigma [7, 26, 27, 29]. In addition, two studies reported that women who were living with fistula were ostracised by their husbands, families and communities [7, 27]. Four studies acknowledged the poor socio-economic status of the women due to their condition [7, 26, 27, 29]. One of the respondents of the included studies [26] in this present review stated: “Customers do not buy my goods anymore”.

Helplessness and sadness were identified as the psycho-social impact of obstetric fistula in only three studies [26, 27, 29]. Also, women with obstetric fistula were found to be depressed due to incontinence in six studies [7, 9, 27–30]. One out of the six studies reported that there was no significant association between depression and age, hence, depression was common across all age groups [30]. They further indicated a significant association between depression and parity where women with obstetric fistula who has given birth five times or more were least depressed.

In addition, women with obstetric fistula were reported to suffer low self-esteem, stigma and discrimination in six studies [3, 7, 9, 26, 27, 29]. Two studies [27, 29] reported that women with obstetric fistula were excommunicated while two studies [9, 26] indicated that women who were diagnosed with obstetric fistula were called names like ‘witch’ and ‘barren woman’. Shame was reported in seven studies [3, 7, 26–29]. Four studies [7, 9, 27, 29] reported perinatal loss among the respondents. Asides stigma from the community, two studies also indicated that some of the women who did not experience stigma, self-stigmatised [3, 29]. Degge et al*.* [3] further reported that successful obstetric fistula repair lessened stigma. Women with obstetric fistula who faced stigma from the community were supported by the family while those that experienced stigma from family members were supported by the community [3]. Likewise, family members of women with obstetric fistula were also reported to experience stigma by association [3].

Feelings of worthlessness were reported in two studies [3, 29]. Women with obstetric fistula were unable to carry out their routine and felt that they were a huge burden to their families [3, 29]. Consequently, these women had suicidal thoughts as reported in Nweke and Igwe [29]. Suicidal thoughts were also identified in two studies [3, 26]. Three studies indicated that women with obstetric fistula had suicidal thoughts as a means of escaping the adverse effects of obstetric fistula [3, 26, 29]. Additionally, two studies reported a loss of identity among women with obstetric fistula [3, 29]. While Nweke and Igwe [29] ascribed the loss of identity to the women’s inability to fulfil their roles in the family and community at large, Degge et al*.* [3] reported three identities assigned to women with obstetric fistula by the society which include “leaking identity” “group identity” (leakers of urine) and “spoiled identity”. However, it was revealed that these identities except the spoiled identity changed due to successful obstetric fistula repair [3].

### Psycho-social support available for women with obstetric fistula in Nigeria

Only five studies reported a form of psycho-social support given to women with obstetric fistula [3, 7, 26, 27, 29]. Three studies reported poor social support from families of the women with obstetric fistula [7, 27, 29]. However, only two studies identified psycho-social support from health workers [27, 29]. Nsemo [26] reported that about 10% of the respondents were empowered. Some respondents received support in form of financial and material provisions from families [27, 29], others received empowerment in the form of vocational training including provisions to begin harnessing their skills [26]. Some women received encouragement and consolation from health workers [27, 29] while others were supported with an avenue to make money since they lacked a source of income [7]. Additionally, Degge et al*.* [3] reported rehabilitation program as psycho-social support offered to women with obstetric fistula.

## Discussion

According to five of the included studies, [3, 7, 9, 26, 27], women with obstetric fistula were faced with either divorce or separation from their spouse. This is similar across several regions in Nigeria as reported in the results. This is probably because as women become incontinent, they are unable to satisfy the sexual urges of their husbands and produce children. Also, they become incapable of performing womanly roles in their households. In Nigeria as well as other African countries, women are expected to be married into families, perform household chores, and produce children [32, 33]. Hence, women unable to produce offspring are not seen as real women and are exempted from community gatherings of women and mothers [32]. Evidence suggests that women who had children before the development of obstetric fistula lived better lives compared to women with obstetric fistula with no children [34, 35]. According to some respondents in one of the selected studies [29], women with obstetric fistula lived with the fear of losing their husbands because they had no children. Most men stay with their wives who had developed obstetric fistula because of their children. These findings are consistent with studies conducted in other developing countries like Kenya [15, 36], Somalia [5], Tanzania [11, 17, 37, 38], Uganda [37, 39, 40], Malawi [12], Ethiopia [41] and Ghana [42].

This present review also revealed that women living with obstetric fistula experience all forms of stigma and discrimination due to their incontinence.. These findings corroborate with findings from studies conducted in Malawi [35], Tanzania [11] and Kenya [15]. In the African setting, infertile and childless women are regarded as a failure to womanhood which attracts stigma to the women in question [43]. Findings from this present review also reveal that these affected women are still discriminated and stigmatised even after undergoing a successful obstetric fistula repair surgery. Similar reports are seen in Kenya [15] and Ethiopia [44]. Also, this present review showed that some of these affected women self-stigmatised. Self-stigmatisation by women with obstetric fistula was also observed in a Ugandan setting [1]. It is possible that those who self-stigmatise do so from general societal norms on personality, character and sickness [45]. Likewise, family members of women with obstetric fistula were reported to be subjected to stigma from this present review. This shows the experiences of stigma by not only the women with obstetric fistula but also the close relations including the spouse. According to Goffman [45], due to stigma by association, an individual may react by ending an existing relationship with the source of the stigma, which is the woman with obstetric fistula in this case. In addition, most cultural norms expect control over bladder and bowel movement, thus the loss of control can attract shame, social isolation and loss of dignity [46–48]. This possibly explains why most women in this present review were socially isolated. Further, evidence suggests a low knowledge and awareness of obstetric fistula in Nigeria [9, 49]. Therefore, people’s actions and reactions towards women with obstetric fistula due to lack of knowledge can result in social isolation of these affected women.

The negative socio-cultural and family experiences Nigerian women with obstetric fistula undergo, leads to the change in the identity of these women. According to Charmaz [50], the comorbidity of obstetric fistula ruptures the social body of the individual, thus such individual undergoes reconstruction on several magnitudes. This present review reveals the different identities women with obstetric fistula are labelled with. Due to incontinence, loss of marriage and inability to perform social activities, women with obstetric fistula lose their identity and roles as wives and women in the community. The several identities identified are in phases. Women with obstetric fistula especially in the Northern region of Nigeria are labelled with the leaking identity which is also known as “mai yoyo” in native Hausa language [3]. However, most women with obstetric fistula transition from the leaking identity to the spoilt identity when they realise how different they have become from other women in the society [3]. At this point, they no longer see themselves as alike to others which are explained by the role identity theory [51]. Hence, these women feel vulnerable, worthless and incomplete. Additionally, some of these women are called names by the members of the community and even family members as reported in this present review. These findings are supported by previous findings from a study in Malawi [12]. This possibly explains why affected women prefer to stay away and isolate themselves from the society.

Also, in some cases, women with obstetric fistula become ostracised as they have lost their cultural identity as revealed in this review. Thus, these women suffer psychological trauma like those observed in Ghana [42], Ethiopia [44], Kenya [15, 36] and Uganda [1], as they cannot live their normal lives with their families. Furthermore, Nigerian women living with obstetric fistula are revealed to suffer from foot drop which make them unable to walk properly without help. As a result, these women are unable to perform their regular activities including activities that bring in income. This was also reported in Ethiopia [44] and Uganda [1, 39]. These women are unable to be gainfully employed and record a decrease in sales of their goods due to incontinence. According to the disability adjusted life years (DALY) evaluation of maternal health burden, women living with obstetric fistula lose most of their years of life due to disability [11, 52]. Most of the women with obstetric fistula in this review were mostly young, hardly acquired formal education and were poor, and obstetric fistula prevented them from gaining employment. As a result, these women are socially marginalised. The social marginalisation of women with obstetric fistula have been reported in other studies as well [10, 11, 53]. Thus, they become poor and beg to survive. This explains the poor socio-economic status observed among these women in this review.

Nigerian women with obstetric fistula are subjected to numerous adverse experiences including lack of support, social stigma and economic incapability which exposes them to various mental health issues such as depression, hopelessness, feelings of worthlessness and loss of dignity as revealed in this present review. Similarly, high rates of depression have been reported in other countries in Sub-Saharan Africa including Niger [54], Tanzania [55] and Kenya [56] which affects their health-seeking behaviour. A study conducted in Ethiopia revealed that nearly all women living with obstetric fistula were depressed when compared to almost two-thirds of patients diagnosed with advanced pelvic organ prolapse [57]. Further, a Tanzanian study reported significant higher rates of depression and post-traumatic stress disorder (PTSD) among women with obstetric fistula compared to other women with other reproductive conditions [17]. Evidence has shown that depression is more common among older women, divorcees and abandoned women living with obstetric fistula [56, 57]. As a result, these women are filled with the thoughts of ending their life of misery. Suicidal thoughts have been reported among women with obstetric fistula in Ethiopia [58] and Niger [54].

### Psycho-social support for obstetric fistula women

Women with obstetric fistula presented high levels of psychological and social suffering which highlights the necessity for psycho-social support. Only five of the included studies reported any form of psycho-social support given to women with obstetric fistula in Nigeria [3, 7, 26, 27, 29]. This present review showed that the type of psychosocial support available to women with fistula, varied. While some received financial support from families, others received empowerment from health workers. This finding concurs to previous findings from studies conducted in Tanzania [17] and Ethiopia [59]. However, studies have showed that the Nigerian Ministry of Health created a National Strategic Framework targeted at eliminating obstetric fistula at all levels within the country [6]. As a result, there are 12 dedicated centres in the country, supported by the state ministries of women’s affairs and social development, offering reintegration and rehabilitation as an important part of obstetric fistula care [60–62]. In addition, the Foundation for Women’s Health Research and Development (FORWARD) and Amref Health Africa deliver programs in African countries including Nigeria, to support women with helpful rehabilitation experiences, health education and community awareness, skill empowerment as well as material gifts [62].

Only one of the included studies [3] reported a rehabilitation program offered to the affected women which made them resilient and hopeful. Also, a review of obstetric fistula in low and middle-income countries reported 80% positive feedback from women receiving rehabilitation services in Nigeria [60]. Similarly, improved emotional wellbeing was observed among women receiving psychosocial counselling and support in South Sudan [63], Kenya, Eritrea, Guinea and Benin [64]. A Tanzanian study reported lower depression score, increased self-esteem, and increased social interaction among Tanzanian women who were provided with social support [65]. Women who feel supported and receive rehabilitation, go ahead to have improved quality of life. Nonetheless, more research is needed to ascertain the impact of the reintegration programs available in Nigeria.

A limitation of this review is that more than half of the included studies were of low quality which could make the quality of this present review questionable. The reliability and relevance of the overall findings of this review as well as the conclusions drawn may be affected [66–68]. Secondly, all included studies were facility-based studies. This indicates that the psycho-social experiences of women with obstetric fistula at home or in the community who have not sought care were left out. This limitation may hinder the transferability of findings in other settings, thus, developing policies and interventions for obstetric fistula women may be difficult in such settings [69]. Also, this highlights the need for more community-based research on obstetric fistula in Nigeria. Nevertheless, this review recommends that research on the social and mental health outcomes of obstetric fistula patients on a large scale should be encouraged. Studies should be done to evaluate the sustainability and reach of the existing psychosocial interventions in Nigeria.

## Conclusion

This review highlights that beyond the clinical and physical outcomes, obstetric fistula women are faced with severe psychosocial outcomes. Hence, psychosocial support is needed to enable full recovery, even after successful repair surgery. Existing psychosocial interventions for obstetric fistula are limited in Nigeria. There is a need for the introduction of more rehabilitation and reintegration programs across the country. This will greatly contribute to the National Strategic Framework for the elimination of obstetric fistula policy goal in Nigeria, as well as the UN Sustainable Development Goal (SDG) 3 by promoting healthy lives and wellbeing of the affected women [70]. Also, more research is needed to evaluate the impact of psycho-social interventions in obstetric fistula care. Focusing on the psycho-social outcomes of obstetric fistula on women in Nigeria would impact other aspects of the SDG goals such as gender equality and women’s empowerment alongside decreasing poverty.

## Data Availability

The datasets used and/or analysed during the current study are available from the corresponding author on reasonable request.

## References

[1] Barageine JK, Beyeza-Kashesya J, Byamugisha JK, Tumwesigye NM, Almroth L, Faxelid E (2015). ”I am alone and isolated”: a qualitative study of experiences of women living with genital fistula in Uganda. BMC Women’s Health.

[2] Wall LL (2006). Obstetric vesicovaginal fistula as an international public health problem. Lancet.

[3] Degge HM, Laurenson M, Dumbili EW, Hayter M. Reflections on identity: narratives of obstetric fistula survivors in North central Nigeria. Qual Health Res [Online] (2019). https://www.ncbi.nlm.nih.gov/pubmed/31578929 10.1177/1049732319877855 31578929

[4] Semere L, Nour MN (2008). Obstetric fistula: living with incontinence and shame. Rev Obstet Gynaecol.

[5] Mohammed AA, Ilesanmi AO, Dairo DM. The experience of women with obstetric fistula following corrective surgery: a qualitative study in Benadir and Mudug regions, Somalia. Obstet. Gynaecol. Int. https://www.hindawi.com/journals/ogi/2018/5250843/10.1155/2018/5250843 PMC6180917 30363732

[6] UNFPA (2019) National strategic frame-work for the elimination of obstetric fistula in Nigeria 2019–2023. https://nigeria.unfpa.org/en/publications/national-strategic-frame-work-elimination-obstetric-fistula-nigeria-2019-2023?page=1

[7] Okoye UO, Emma-Echiegu N, Tanyi PL. Living with vesico-vaginal fistula: experiences of women awaiting repairs in Ebonyi state, Nigeria. Tanzania J Health Res*.* 16(4) 10.4314/thrb.v16i4.9 26891522

[8] Bashah DT, Worku AG, Mengistu MY (2018). Consequences of obstetric fistula in sub-Sahara African countries, from patients’ perspective: a systematic review qualitative studies. BMC Women’s Health.

[9] Emma-Echiegu N, Okoye UO, Odey ES (2014). Knowledge of causes of VVF and discrimination suffered by patients in Ebonyi state, Nigeria: a qualitative study. Social Work Public Health.

[10] Ahmed S, Holtz SA (2007). Social and economic consequences of obstetric fistula: life changed forever?. Int J Gynaecol Obstet.

[11] Mselle LT (2011). “I am nothing”: experiences of loss among women suffering from severe birth injuries in Tanzania. BMC Women’s Health.

[12] Changole J, Thorsen VC, Kafulafula U (2017). “I am a person but I am not a person”: Experiences of women living with obstetric fistula in the central region of Malawi. BMC Pregnancy Childbirth.

[13] Biadgilig S, Lakew Y, Reda AA, Deribe K (2013). A population-based survey in Ethiopia using questionnaire as proxy to estimate obstetric fistula prevalence: results from demographic and health survey. Reprod Health.

[14] Amodu OC, Salami B, Richter S (2017). Obstetric fistula and sociocultural practices in Hausa community of Northern Nigeria. Women Birth.

[15] Khisa AM, Nyamongo IK (2012). Still living with fistula: an exploratory study of the experience of women with obstetric fistula following corrective surgery in West Pokot, Kenya. Reprod Health Matters.

[16] Goh JTW (2005). Mental health screening in women with genital tract fistulae. Int J Obstet Gynaecol.

[17] Wilson SM (2015). Psychological symptoms among obstetric fistula patients compared to gynaecology outpatients in Tanzania. Int J Behav Med.

[18] WHO. Obstetric fistula: Guiding principles for clinical management and programme development (2006). https://apps.who.int/iris/handle/10665/43343 10.1016/j.ijgo.2007.06.032 17880979

[19] Ijaiya MA, Rahman AG, Aboyeji AP, Olatinwo AW, Esuga SA, Ogah OK, Raji HO, Adebara IO, Akintobi AO, Adeniran AS, Adewole AA (2010). Vesicovaginal fistula: a review of Nigerian experience. West Afr J Med.

[20] Oluwasolaa TAO, Bello OO (2020). Clinical and psychosocial outcomes of obstetric fistulae in Sub-Saharan Africa: a review of literature. J Basic Clin Reprod Sci.

[21] Njoku CO, Njoku AN (2011). Obstetric fistula: the agony of unsafe motherhood. a review of Nigeria experience. J Adv Med Med Res.

[22] Hannes K. Chapter 4: Critical appraisal of qualitative research. In: Noyes J, Booth A, Hannes K, Harden A, Harris J, Lewin S, Lockwood C (editors), *Supplementary Guidance for Inclusion of Qualitative Research in Cochrane Systematic Reviews of Interventions.* Version 1 (updated August 2011). Cochrane Collaboration Qualitative Methods Group, 2011. http://cqrmg.cochrane.org/supplemental-handbook-guidance

[23] Bergs J (2015). Barriers and facilitators related to the implementation of surgical safety checklists: a systematic review of the qualitative evidence. BMJ Qual Saf.

[24] Taylor N (2015). High performing hospitals: a qualitative systematic review of associated factors and practical strategies for improvement. BMC Health Serv Res.

[25] Alberski W (2012). Selected functions of narrative structures in the process of social and cultural communication. Styles Commun.

[26] Nsemo AD (2014). ‘Influence of abandonment, stigmatization and social isolation on the coping strategies of women with vesico vaginal fistula in Akwa Ibom state. Nigeria’.

[27] Fehintola AO (2019). ‘Birth and sorrow: the medico-social consequences of obstetric fistula in Ilesha, Nigeria. Trop J Obstet Gynaecol.

[28] Kabir M, Iliyasu Z, Abubakar JS, Umar UI (2003). Medico-social problems of patients with vesico-vaginal fistula in Muritala Mohammed specialist hospital, Kano. Ann Afr Med.

[29] Nweke DN, Igwe MN (2017). Psychosocial experiences of subjects with vesicovaginal fistula: a qualitative study. Global J Med Public Health.

[30] Hassan M, Nasir S (2019). ‘Co morbidities associated with vesico vaginal fistula in patients managed in Maryam Abacha Fistula Hospital Sokoto, North-western Nigeria. Trop J Obstet Gynaecol.

[31] Critical Appraisal Skills Programme (CASP) (2018) CASP checklists. https://casp-uk.net/casp-tools-checklists/

[32] Dyer SJ. The value of children in African countries–Insights from studies on infertility. https://www.tandfonline.com/doi/full/10.1080/01674820701409959?scroll=top&needAccess=true (2007) 10.1080/01674820701409959 17538814

[33] Chimbatata NBW, Malimba C (2016). ‘Infertility in Sub-Saharan Africa: a woman’s issue for how long? A qualitative review of literature. Open J Soc Sci.

[34] Turan JM, Johnson K, Polan ML (2007). Experiences of women seeking medical care for obstetric fistula in Eritrea: implications for prevention, treatment, and social reintegration. Int J Res Policy Pract.

[35] Yeakey MP (2009). The lived experience of Malawian women with obstetric fistula. Cult Health Sex.

[36] Kimani ZM, Ogutu O, Kibe A (2014). The prevalence and impact of obstetric fistula on women of Kaptembwa-Nakuru, Kenya. Int J Sci Technol.

[37] Bangser M (2011). Childbirth experiences of women with obstetric fistula in Tanzania and Uganda and their implications for fistula program development. Int Urogynaecol J.

[38] Mselle LT, Kohi TW (2015). Living with constant leaking of urine and odour: thematic analysis of socio-cultural experiences of women affected by obstetric fistula in rural Tanzania. BMC Women’s Health.

[39] Kabayambi J (2014). Living with obstetric fistula: Perceived causes, challenges and coping strategies among women attending the fistula clinic at Mulago hospital, Uganda. Int J Trop Disease Health.

[40] Bomboka BJ, N-Mboowa MG, Nakilembe J (2019). Post-effects of obstetric fistula in Uganda: a case study of fistula survivors in KITOVU mission hospital (MASAKA), Uganda. BMC Public Health.

[41] Tollosa DN, Kibret MA (2013). Causes and consequences of obstetric fistula in Ethiopia: a literature review. Int J Med Res Health Sci.

[42] Mwini-Nyaledzigbor PP, Agana AA, Pilkington FB. Lived experiences of Ghanaian women with obstetric fistula (2013). https://www.tandfonline.com/doi/abs/10.1080/07399332.2012.755981 10.1080/07399332.2012.755981 23641897

[43] Van Balen F, Bos HMW (2009). The social and cultural consequences of being childless in poor-resource areas. Facts Views Vis Obstet Gynaecol.

[44] Gebresilase YT (2014). ‘A qualitative study of the experience of obstetric fistula survivors in Addis Ababa, Ethiopia. Int J Women’s Health.

[45] Goofman E. Stigma: notes on the management of spoiled identity (2009). https://books.google.co.uk/books?id=zuMFXuTMAqAC&lr=&source=gbs_navlinks_s

[46] Weinberg MS, Williams CJ (2005). Faecal matters: Habitus, embodiments, and deviance. Soc Probl.

[47] Norton NJ. Impact of faecal and urinary incontinence on the health consumer: Barriers on diagnosis and treatment—A patient perspective (2007). https://consensus.nih.gov/2007/incontinenceabstracts.pdf#page=47

[48] Mota DM, Barros AJD (2008). Toilet training: methods, parental expectations and associated dysfunctions. J Pediatria.

[49] Umoiyoho AJ, Inyang-Etoh EC (2012). Community misconception about the aetiopathogenesis and treatment of vesico-vaginal fistula in Northern Nigeria. Int J Med Biomed Res.

[50] Charmaz K. Experiencing chronic illness (2000). https://books.google.co.uk/books?hl=en&lr=&id=YISsqYc72FoC&oi=fnd&pg=PA277&dq=Charmaz,+2000&ots=Isek3wwE9B&sig=0LPxf1Iu1QU_7tE5YFwjUoKPGv0#v=onepage&q=Charmaz%2C%202000&f=false

[51] Stets JE, Burke PJ (2000). Identity theory and social identity theory. Soc Psychol Q.

[52] Guyatt GH, Cook DJ. Health status, quality of life, and the individual (1994). https://jamanetwork.com/journals/jama/article-abstract/378382 8057520

[53] Wall LL (2005). The obstetric vesicovaginal fistula in the developing world. Obstet Gynaecol Surv.

[54] Alio AP (2011). The psychosocial impact of vesico-vaginal fistula in Niger. Arch Gynaecol Obstet.

[55] Siddle K (2013). Psychosocial impact of obstetric fistula in women presenting for surgical care in Tanzania. Int Urogynaecol J.

[56] Weston K (2011). Depression among women with obstetric fistula in Kenya. Int J Obstetr Gynaecol.

[57] Zeleke BM (2013). Depression among women with obstetric fistula and pelvic organ prolapse in northwest Ethiopia. BMC Psychiatry.

[58] Muleta M, Rasmussen S, Kiserud T (2010). Obstetric fistula in 14,928 Ethiopian women. Acta Obstetricia et Gynecologica.

[59] Browning A, Menber B (2008). Women with obstetric fistula in Ethiopia: a 6-month follow up after surgical treatment. Int J Obstet Gynaecol.

[60] Capes T (2011). Obstetric fistula in low- and middle-income countries. Mt Sinai J Med.

[61] Shittu OS (2007). A review of postoperative care for obstetric fistulas in Nigeria. Int J Gynecol Obstet.

[62] Shallon A (2018). Social reintegration and rehabilitation of obstetric fistula patients before and after repair in sub-Saharan Africa: a systematic review. NJOG.

[63] Ojengbede OA (2014). Group psychological therapy in obstetric fistula care: a complementary recipe for the accompanying mental ill health morbidities?. Afr J Reprod Health.

[64] El Ayadi AM (2020). Rehabilitation and reintegration programming adjunct to female genital fistula surgery: A systematic scoping review. Int J Gynecol Obstet.

[65] Dennis AC (2016). Experiences of social support among women presenting for obstetric fistula repair surgery in Tanzania. Int J Women’s Health.

[66] Rees K, Ebrahim S (2001). Promises and problems of systematic reviews. Heart Drug.

[67] Garg AX, Hackam D, Tonelli M (2008). Systematic review and meta-analysis: when one study is just not enough. Clin J Am Soc Nephrol.

[68] Yuan Y, Hunt RH (2009). Systematic reviews: the good, the bad, and the ugly. Am J Gastroenterol.

[69] Anderson C (2010). Presenting and evaluating qualitative research. Am J Pharm Educ.

[70] UN Sustainable Development Goals (2020). https://www.un.org/sustainabledevelopment/sustainable-development-goals/

[71] Ahmed S, Anastasi E, Laski L (2016). Double burden of tragedy: stillbirth and obstetric fistula. Lancet.

[72] Landry E, Frajzyngier V, Ruminjo J, Asiimwe F, Hamidou B, Bello A, Danladi D, Oumarou SG, Idris S, Inoussa M, Kanoma B, Lynch M, Mussell F, Podder DC, Wali A, Mielke E, Barone MA (2013). Profiles and experiences of women undergoing genital fistula repair: findings from five countries. Glob Public Health.

[73] UNFPA (2014) Annual report. https://www.unfpa.org/sites/default/files/pub-pdf/UNFPA_annual_report_2014_en.pdf

[74] Bello OO, Morhason-Bello IO, Ojengbede OA (2020). (2020) ‘Nigeria, a high burden state of obstetric fistula: a contextual analysis of key drivers’. Pan Afr Med J.

[75] Maheu-Giroux M (2016). Risk factors for vaginal fistula symptoms in Sub-Saharan Africa: a pooled analysis of national household survey data. BMC Pregnancy Childbirth.

[76] Donnay F, Weil L (2004). Obstetric fistula: the international response. Lancet.

[77] Meyer L (2007). Commonalities among women who experienced vesicovaginal fistulae as a result of obstetric trauma in Niger: Results from a survey given at the National Hospital Fistula Center, Niamey, Niger. Am J Obstetr Gynaecol.

[78] Norman AM, Breen M, Richter HE (2007). Prevention of obstetric urogenital fistulae: some thoughts on a daunting task. Int Urogynaecol J Pelvic Floor Dysfunct.

[79] Roush KM (2009). Social implications of obstetric fistula: An integrative review. J Midwifery Women’s Health.

[80] Pope R, Bangser M, Requejo JH (2011). ‘Restoring dignity: Social reintegration after obstetric fistula repair in Ukerewe. Tanzania, Global Public Health.

[81] Melah GS (2007). Risk factors for obstetric fistulae in North-Eastern Nigeria. J Obstet Gynaecol.

[82] Erulkar AS, Bello M. The experience of married adolescent girls in Northern Nigeria (2007). https://www.ohchr.org/Documents/Issues/Women/WRGS/ForcedMarriage/NGO/PopulationCouncil24.pdf

[83] Women’s Dignity Project and Engender Health. Risk and Resilience: Obstetric fistula in Tanzania. https://books.google.co.uk/books/about/Risk_and_resilience.html?id=0uoPAQAAMAAJ&redir_esc=y

[84] Murphy M. Social consequences of vesico-vagina fistula in Northern Nigeria (1981). https://www.cambridge.org/core/journals/journal-of-biosocial-science/article/social-consequences-of-vesicovaginal-fistula-in-northern-nigeria/DB5E0AF4CBEC504AC72800D39823C173 10.1017/s0021932000013304 7287771

